# A novel and simple formula to predict liver mass in porcine experimental models

**DOI:** 10.1038/s41598-019-48781-2

**Published:** 2019-08-28

**Authors:** Lilia Martínez de la Maza, Verónica Prado, Amelia J. Hessheimer, Javier Muñoz, Juan Carlos García-Valdecasas, Constantino Fondevila

**Affiliations:** 1 Hepatopancreatobiliary Surgery & Transplantation, General & Digestive Surgery Service, Institut Clínic de Malalties Digestives i Metabòliques (ICMDM), Hospital Clínic, CIBERehd, IDIBAPS, University of Barcelona, Barcelona, Spain; 2 Hospital de Mollet, Fundació Sanitaria Mollet, Mollet del Vallès, Spain

**Keywords:** Liver, Experimental models of disease

## Abstract

A primary limitation in hepatic surgery is leaving a remnant liver of adequate size and function. Experimental models have been designed to study processes of liver injury and regeneration in this context, yet a formula to accurately calculate liver mass in an animal model is lacking. This study aims to create a novel and simple formula to estimate the mass of the native liver in a species of pigs commonly used in experimental liver surgery protocols. Using data from 200 male weanling Landrace-Large White hybrid pigs, multiple linear regression analysis is used to generate the formula. Clinical features used as variables for the predictive model are body mass and length. The final formula for pig liver mass is as follows: Liver mass (g) = 26.34232 * Body mass (kg) – 1.270629 * Length (cm) + 163.0076; R^2^ = 0.7307. This formula for porcine liver mass is simple to use and may be helpful in studies using animals of similar characteristics to evaluate restoration of liver mass following major hepatectomy.

## Introduction

In recent decades, the application of liver resection has expanded in its indication and application. However, a primary limiting factor for performing major liver resection in the clinical setting remains the need to leave a liver remnant of adequate size, quality, and function, in order to avoid the development of post-hepatectomy liver failure (PHLF)/“small-for-size” syndrome (SFSS) in the postoperative period. PHLF/SFSS is characterized by progressive cholestasis, coagulopathy, encephalopathy, ascites, gastrointestinal bleeding, and/or renal failure. Once PHLF/SFSS is diagnosed, it is associated with high patient morbidity and mortality^1,2^.

While the pathophysiology of PHLF/SFSS is multifactorial, the size of the remnant liver is a critical factor in its development. Depending on the conditions of the patient and the liver itself, more or less remnant liver mass is needed to avoid PHLF/SFSS. Typically, it is recommended that the ratio between the remnant liver and recipient body mass be >0.8–1% and the remnant represent >25–30% of native liver mass for normal livers or >40% in livers that are cholestatic, cirrhotic, steatotic, or injured by chemotherapy^3,4^. Therefore, to be able to adequately evaluate the remnant liver mass preoperatively, is of great interest.

Different animal models have been developed to study liver resections. Our research group has worked extensively in experimental models of orthotopic liver transplantation in pigs^5–11^. Pigs were selected because they present similar liver size, anatomy, and physiology to that of humans. We have also developed a porcine model of extended liver resection, in which we evaluate postoperative liver function, regeneration, and survival. In the latter model, we analyze the impact of the remnant liver mass on recovery and regeneration following major hepatectomy. While we can know the mass of the liver that is resected, we cannot easily know the true mass of the liver that is left behind. Therefore, the aim of the present study is to create a formula to estimate total liver mass using data from our previous experience with total hepatectomies performed in the context of porcine liver transplantation.

## Patients and Methods

### Animals

Since 2005, our group has performed total hepatectomy in over 200 weanling (2–4 months) male Landrace-Large White hybrid pigs^5–11^. Animals included in this study were from the same provider, underwent through equal transportation, had the same access to and type of food as to water, and were subjected to the same environmental acclimation and perioperative care. As well, all animals had a certificate of good health signed by the animal provider.

Procedures were conducted in the Animal Experimentation Unit at the University of Barcelona Medical School and in accordance with current national and European regulations. The Animal Experimentation Unit is authorized by the Catalan Department of Agriculture, Husbandry, and Fisheries (authorization number B9900020), registered in the General Registry of Livestock Facilities (ES080190036536), and accredited by the International Standards Organization (ISO 9001:2015). Animals were cared for according to the guidelines of the University of Barcelona Committee on Ethics in Animal Experimentation and the Catalan Department of the Environment Commission on Animal Experimentation.

### Statistical analysis

Data from 200 pigs was used in this study. Using randomized split sample technique^12,13^, 142 pigs (70% of the overall sample) were included in the derivation group and 58 (30%) in the validation group. The following variables were evaluated in the derivation group: body mass (kg), snout-to-rump length (cm), and body surface area (m2). Variables associated with total liver mass on univariate analysis (P < 0.2) were selected for the initial models. Using the allsets tool, seven initial models were generated^14,15^. The final model was selected based on Mallows’ Cp and adjusted R2 values, as high adjusted R2 is essential for good predictive-model performance^16^. The selected model was then validated in the validation group. Differential loss (shrinkage value) <10% was deemed necessary to consider the model valid and reliable.

Results are presented as frequencies and percentages for categorical variables and median and interquartile range for continuous variables. For univariate analyses, Chi-square test was used for categorical variables, Student’s *t* test or ANOVA for normal continuous variables, and Mann-Whitney or Kruskal Wallis tests for non normally distributed continuous variables. In all statistical analyses, significance was set at P < 0.05. All data analysis was performed using STATA version 4.0 (StataCorp LLC, College Station, Texas, USA).

### Ethical approval

This study received ethical approval by the University of Barcelona Committee on Ethics in Animal Experimentation and the Catalan Department of the Environment Commission on Animal Experimentation. All experiments were performed in accordance with current national and European regulations.

## Results

The characteristics and features of both samples cohorts are described in Table 1. No significant differences between the two groups were detected.

**Table 1:** Characteristics of Porcine Sample Groups.

	Derivation Group (N = 142)	Validation Group (N = 58)	*P*
Body mass (kg)			
Mean	27.49	26.41	0.231
Median	29.00	29.00	
25–75% IQR	21–33	21.50–31	
Length (cm)			
Mean	97.68	96.33	0.183
Median	98.00	97.50	
25–75% IQR	92–105	88.75–103	
BSA			
Mean	6388	6248	0.285
Median	6683	6683	
25–75% IQR	5408–7293	5492–7001	

IQR, interquartile range.

**Figure 1:**
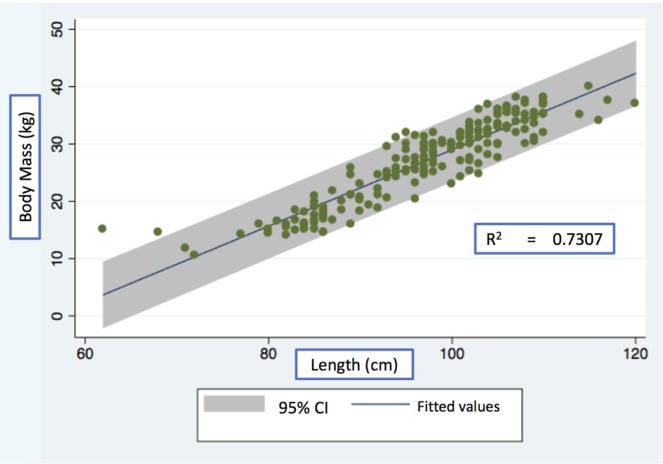
Linear regression model scatter plot with corresponding 95% confidence interval.

**Figure 2:**
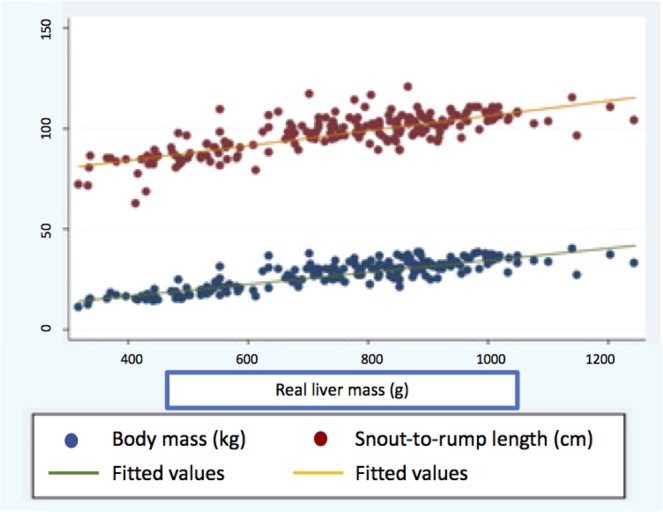
Scatter plots depicting correlations between the individual predictors and total liver mass.

On univariate analysis, body mass, snout-to-rump length, and BSA were all significantly associated with total liver mass. These three variables were introduced in the allsets tool to determine all possible equations for predicting porcine liver mass, and seven different models were identified. Ultimately, body mass and snot-to-rump length were selected as the final predictors. After performing multiple linear regression analysis, the final formula for pig liver mass (PLM, g) was generated: 26.34232 * Body mass (kg) – 1.270629 * Length (cm) + 163.0076; R^2^ = 0.7307 and adjusted R^2^ = 0.7268, Mallows’ Cp 10.28, variance inflation factor (VIF) 6.2 (Fig. 1). For the variables used, Fig. 2 depicts the individual correlations between both of the predictors and total liver mass.

**Figure 3:**
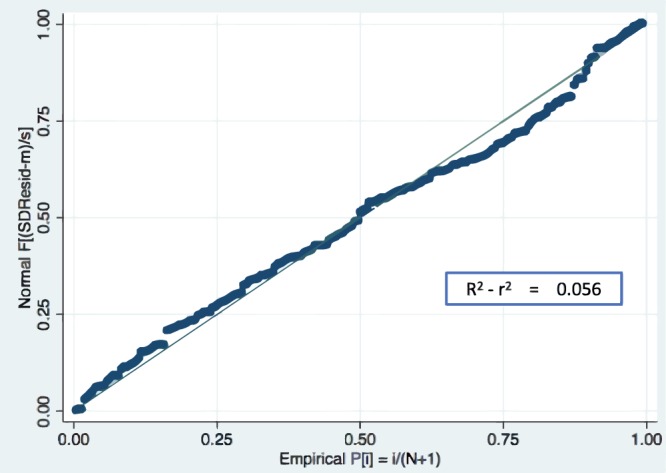
Regression model residuals plot. No significant variation of residuals was detected.

The formula was validated in a split group consisting in 30% of the subjects in the original sample. Differential loss of prediction (shrinkage) was 5.56% (R^2^-r^2^Loss = 0.05645025). To further validate our model, we calculated the variance of the residuals of the multiple regression analysis and did not find any significant variation of residuals (Fig. 3).

**Figure 4:**
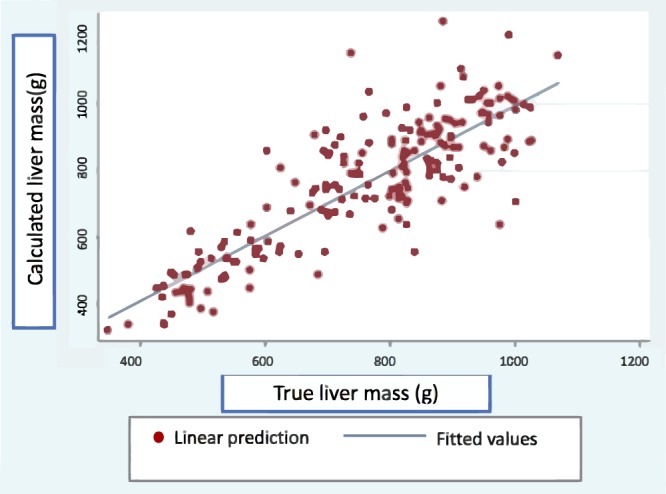
True and calculated liver mass scatter plot.

Finally, using the new pig liver mass formula, we used the entire sample to compare true versus calculated liver mass (Table 2 & Fig. 4).

**Table 2:** True vs. Calculated liver mass.

	Derivation Group (N = 142)	Validation Group (N = 58)	*P*
True Liver Mass (g) (TLM)			
Mean	763.01	737.02	0.399
Median	793.5	753.5	
IC 25–75	584.25–918	583.75–868	
Calculated Liver Mass (g) (CLM)			
Mean	763	736.21	0.318
Median	803.68	806.68	
IC 25–75	603.11–899.84	619.45–849.38	
Difference between TLM and CLM (g)			
Mean	−0.0001	0.8	0.961
Median	−8.82	16.13	
IC 25–75	−65.5–56	−63.98–49.24	

IQR, interquartile range.

## Discussion

In liver surgery, accurate calculation of remnant liver size is critically important to reducing postoperative morbidity and mortality, in particular due to PHLF/SFSS^17^. To this end, several formulae have been developed to estimate liver mass or volume in humans preoperatively^18,19^. Such formulae typically include clinical features, such as body mass, height, and/or body surface area (BSA)^20^. The formula proposed by Kokudo *et al*. uses more specific morphological features, namely thoracic and abdominal width^21^. All of these formulae have been created by comparing estimated size with either volumetric calculation obtained through computed tomography (CT) scanning or real liver mass obtained in the context of whole or partial liver transplantation. The overall accuracy and applicability of the aforementioned formulae may be limited to a certain extent by patient gender, age, and race. Also, BSA may be calculated in different manners, and formulae that include BSA as an input variable may vary accordingly.

While several formulae are available for liver size prediction in humans, little is available for studies in animals. Experimental models are of critical importance for surgical investigation, as they offer the opportunity to test novel techniques and therapies under relatively stable conditions prior to their application in humans. Animal studies on liver regeneration are particularly relevant in that they can help elucidate mechanisms of liver regeneration *in vivo* and can be used in the extreme to simulate and attempt to prevent and/or treat pathological regenerative processes^22^.

Until now, assessment of total and remnant liver size in porcine liver resection studies has generally required the use of advanced imaging techniques, such as CT or magnetic resonance imaging^23^. Bekheit *et al*. recently published an assessment of porcine liver anatomy in which volumetric characteristics as well as features of the vascular and biliary trees were described using CT performed on 37 female pigs^24^. While cross-sectional imaging is relatively reliable, especially in clinical practice, it is costly and not always easily accessible for use in animals.

We wanted to create a formula for predicting porcine liver mass that was simple to use and that incorporated morphological variables that were easy to obtain. We chose not to include BSA, given that there is no universally accepted formula for calculating BSA nor, for that matter, one that has been validated for pigs. The formula that we have created, based on body mass and length, appears to estimate total liver mass well, with R^2^ = 0.73 and differential loss of prediction <10% (5.56%) on external validation. Nonetheless, the study does have limitations. Animals included in the study were all males of common European pig breeds (hybrid Landrace-Large White) and aged between two and four months. This formula needs to be tested in a wider variety of pigs of different ages, breeds, and sex to determine whether it remains accurate.

The pig is the most commonly selected large animal for performing pre-clinical studies on the liver. Pigs are robust and readily available; their livers present a similar size to those of humans and can also be divided into eight segments based on vascular supply and biliary drainage^25^. An important anatomical aspect of the porcine liver that distinguishes it from that of other species is its intimate relationship with the inferior vena cava (IVC). The porcine IVC is contained within and cannot be separated from the tissue of the caudate and right lateral lobes. As such, the minimum remnant volume after major hepatic resection is greater in pigs than in other species. Different authors have evaluated the percentage of overall liver mass that each liver segment represents, and the caudate and complete right lateral lobes appear to constitute roughly 30% of the entire porcine liver^25–27^. Using our formula, however, our hope is that future estimates of the remnant liver in major hepatectomy studies in pigs will be more accurate than those based on rough percentages.

In summary, we have created a novel formula to predict pig liver mass in a simple and reproducible manner. This formula should be a useful tool for future liver surgery studies performed in the porcine model.

## Data Availability

The data generated and analyzed during this study are available from the corresponding author on reasonable request.
